# Nucleus of the solitary tract regulation of cue-induced appetitive behaviors via midbrain dopamine neurons

**DOI:** 10.1038/s41386-026-02417-y

**Published:** 2026-04-22

**Authors:** Jo Ann Yap, Yisha Helen Gu, Colin Vuong, John M. Power, Zhi Yi Ong

**Affiliations:** 1 School of Psychology, University of New South Wales, UNSW, Sydney, NSW Australia; 2 Translational Neuroscience Facility and Department of Physiology, School of Biomedical Sciences, University of New South Wales, UNSW, Sydney, NSW Australia

**Keywords:** Feeding behaviour, Reward

## Abstract

Internal states including stress and satiety, affect cue-induced feeding behaviors, yet the underlying neural mechanisms remain poorly understood. The nucleus of the solitary tract (NTS), a key hindbrain hub that integrates interoceptive and viscerosensory signals and projects to reward processing nuclei, is anatomically well-positioned to modulate cue-induced feeding behaviors in response to internal state changes. Using behavioral paradigms combined with chemogenetics and fibre photometry, this study investigated the hypothesis that NTS mediates the effects of stress and satiety on cue-induced feeding behaviors via A2 neurons and modulation of ventral tegmental area (VTA) dopamine signaling. We first showed that both foot shock stress and outcome specific satiety (i.e., sucrose prefeed) reduced cue-induced appetitive behavior. Inhibition of NTS neurons only attenuated the suppressive effect of foot shock stress, but not that of outcome specific satiety, indicating that NTS is required for stress-induced suppression of cue-induced appetitive behavior. Further investigation into the contributing neural phenotype revealed that stimulation of NTS A2 neurons reduced conditioned approach and suppressed cue-evoked VTA dopamine neural activity, without any effects on lateral hypothalamus (LH) neuron activity. Together, these findings suggest that NTS neurons mediate the effects of foot shock stress, but not outcome specific satiety, on cue-induced appetitive behavior, in part through activation of NTS A2 neurons and modulation of cue-evoked VTA dopamine neural activity. These results provide an NTS-mediated mechanism through which stress suppresses cue-induced feeding behavior.

## Introduction

Food cues are potent drivers of eating. Through Pavlovian conditioning, neutral cues paired with food become conditioned stimuli that predict food availability. These cues elicit a range of conditioned responses from behavioral (e.g., craving) to physiological (e.g., salivation), and gain incentive salience, which triggers the desire to seek out and consume food [1, 2]. This is referred to as cue-induced feeding.

The ability to regulate cue-induced feeding behaviors in response to changes in internal states and environmental demands is critical for survival. When under threat or during stressful situations, behavioral responses to food cues are significantly attenuated. For example, in rodents, chronic social defeat stress or acute foot shock stress suppresses cue-induced approach or motivated behaviors [3, 4]. Similarly, satiety also suppresses responses to food cues. When food rewards are devalued through outcome-specific satiety (i.e., a process in which consumption of a particular food reduces the motivation to consume that same food, without affecting the consumption of other foods/outcomes), animals show reductions in cue-induced appetitive responding for the devalued food reward [5, 6]. While both stress and satiety can suppress cue-induced feeding behaviors, whether they engage shared or distinct neural mechanisms remains unclear.

The nucleus of the solitary tract (NTS), a key hindbrain nucleus known to integrate interoceptive and viscerosensory information, is well-positioned to modulate feeding behaviors in response to internal states. For example, NTS neurons are responsive to both stress and metabolic signals: psychogenic stressors activate caudal NTS A2 neurons [7, 8] and inhibition of NTS preproglucagon (PPG) neurons prevents restraint stress-induced food intake suppression [9]. Similarly, gut nutrient infusion activates NTS A2, PPG, proenkephalin and leptin receptor-expressing neurons and stimulation of NTS A2 and PPG neurons suppresses food intake [10–12]. While it is well-established that NTS neurons respond to internal signals to regulate food intake, whether they also integrate internal signals to affect feeding behaviors driven by environmental cues is unknown.

Emerging evidence supports a role for NTS neurons in regulating cue-induced feeding behaviors. NTS leptin receptor and glucagon-like peptide-1 (GLP-1) receptor signaling prevents conditioned place preference for a sucrose reward [13, 14], and oxytocin delivery to the NTS suppresses cue-induced reinstatement of food-seeking [15]. Both GLP-1 and oxytocin activate NTS A2 neurons, which implicates a role for these neurons in suppressing conditioned reward responses. Supporting this, we previously showed that noradrenaline binding in the nucleus accumbens (NAc), which receives its primary noradrenergic input from NTS A2 neurons [16], declines during food cue presentation and conditioned approach [17].

Furthermore, NTS A2 neurons project to brain regions important in food cue processing and motivation, including the ventral tegmental area (VTA) and lateral hypothalamus (LH) [11], thus indicating possible circuits through which NTS regulates cue-induced feeding behaviors. Together, these findings suggest that the NTS may integrate internal state signals to modulate cue-driven behaviors.

In this study, we examined whether and how NTS neurons regulate cue-induced feeding behaviors in response to stress and satiety, focusing on the contribution of A2 neurons and downstream VTA dopamine neural activity. We found that while both foot shock stress and outcome specific satiety suppressed cue-induced feeding, inhibition of NTS neurons only attenuated the suppressive effect of stress, but not satiety, indicating that NTS is required for stress-induced suppression of cue-induced appetitive behavior. Similar to stress, activation of NTS A2 neurons reduced cue-induced appetitive behavior and this activation also suppressed food cue-evoked VTA dopamine neuron activity. These findings suggest an NTS-mediated mechanism by which stress suppresses cue-induced feeding behaviors.

## Materials and methods

### Animals

Male TH Cre Sprague Dawley rats (SD-TH-Cre^tm1sage^, Sage Laboratories, Cambridge, United Kingdom) and wild type Sprague Dawley rats (Ozgene, Western Australia) were 300–400 g upon arrival. Animals were group-housed in cages with ventilated racks located in a climate-controlled colony room with a 12 h light/dark cycle (lights off at 7:00 pm). Chow and water were available *ad libitum* unless otherwise stated. All experimental procedures were conducted in the light cycle and were approved by the University of New South Wales Animal Care and Ethics Committee.

### Surgery

Rats were anaesthetized with a mixture of 1.3 ml/kg ketamine (Ketamil 100 mg/ml, Ilium) and 0.3 ml/kg xylazine (Xylazil 20 mg/ml, Ilium) injected intraperitoneally (i.p). A subcutaneous (s.c) injection of 0.5% Bupivacaine (Cenvet) was also administered to the incision site as a local anesthetic. Viruses were infused at a rate of 100 nL/min. Jeweler screws and dental cement were used to secure the cannula to the skull. Rats were left to recover for one week post-surgery. See Supplementary information for viral vectors used.

### *Pavlovian appetitive conditioning*

To assess cue-induced feeding behaviors, we used the Pavlovian Appetitive Conditioning paradigm (Details in Supplementary information). To determine whether NTS A2 neurons affect cue-induced appetitive behavior, TH Cre rats injected with hM3Dq (*n* = 5) or eGFP (reporter control; *n* = 8) to the NTS were given i.p. injections of either vehicle (5% DMSO, 95% Saline) or CNO (1 mg/kg) 45 min prior to test, as previously described [11]. To quantify cue-induced appetitive behavior, elevation scores were calculated as the difference between the number of magazine entries during the 15 s CS + /− period and the number of magazine entries during the 15 s pre-CS + /− period, divided by the number of cue presentations.

### Foot shock stress

To determine whether stress suppresses cue-induced appetitive behavior, rats (*n* = 11) received 0.6 mA foot shocks (0.5 s, with an ITI of 1 min) for 30 min in the same apparatus as Pavlovian behavioral training. Immediately following foot shock session, rats were tested on the Pavlovian Appetitive Conditioning paradigm. To examine whether NTS mediates the effects of stress on cue-induced appetitive behavior, rats with NTS hM4Di (*n* = 8) received i.p injection of either vehicle or CNO (5 mg/kg) 30 min prior to foot shock session and tested after receiving foot shocks. A ‘no foot shock’ condition was also included prior to test days, where rats were placed in the chambers for testing without receiving foot shocks. Treatments were counterbalanced within-subjects, with a minimum of 2 days in between test days.

### Sucrose prefeed

To determine whether devaluing the reward outcome suppresses cue-induced appetitive behavior, a separate group of rats (*n* = 10) were fed with sucrose pellets to satiety prior to test. There were three conditions: ad libitum, sucrose prefeed and food deprived. In ‘ad libitum’ and ‘sucrose prefeed’ conditions, rats were given chow and water ad libitum in their home cages. In the ‘food deprived’ condition, chow was removed from home cages for 23 h and returned after test session, while water was available *ad libitum*. This experiment was conducted using a within-subjects design such that all rats were tested in all 3 conditions. During test day, rats in the ‘ad libitum’ and ‘food deprived’ conditions were placed in the chambers and behaviors assessed during the Pavlovian Appetitive Conditioning paradigm. Rats in the ‘prefeed’ condition were given 10 min access to sucrose pellets in the conditioning chamber to consume to satiety prior to the Pavlovian test. To ensure that the effects were specific to the reward outcome (i.e., sucrose), a separate group of rats (*n* = 17) were food deprived and refed with chow 30 min prior to test.

To determine whether NTS neurons mediate the effects of sucrose prefeed on cue-induced appetitive behavior, rats (*n* = 8) with hM4Di in the NTS received i.p injection of either vehicle or 5 mg/kg CNO 45 min prior to Pavlovian test session. Rats were either sated (‘ad libitum’ condition) or pre-fed with sucrose (‘sucrose prefeed’ condition) 10 min prior to test. This was a within-subjects, counterbalanced design, with treatments separated by at least 2 days.

### Fiber photometry

Fiber photometry recordings were performed using the Fiber Photometry Systems from Doric Lenses and Tucker Davis Technologies (RZ5P, Synapse) to measure VTA dopamine neural activity during Pavlovian Appetitive Conditioning. TH Cre rats (*n* = 10) injected with hM3Dq in NTS and GCaMP6f in VTA had their fiber optic cannula attached to a 0.39 NA, Ø400 μm core patch cord. Rats were habituated to tethered patch cords prior to recording. During test days, rats were injected with vehicle or CNO (1 mg/kg) 45 min before test. (Details of fiber photometry methods and analysis in Supplementary information).

### Cannula and viral placement verification

Rats were injected with Lethabarb (diluted 1:1 in saline) and transcardially perfused with 4% paraformaldehyde. Brains were extracted, post-fixed overnight and transferred into 20% sucrose in PBS the following day. Coronal sections of NTS, VTA and LH were collected at 35 µm using a cryostat (Leica Microsystems, CM1950) and stored in PBS with 0.1% Sodium Azide. For cannula placement verification, brain slices were sectioned and mounted directly onto a slide. Sections were then visualized using a light microscope to identify placement of cannula. See Supplementary information for methods on fluorescent immunohistochemistry.

### Statistical analysis

Data were expressed as mean ± SEM and analyzed using IBM SPSS Statistics 29 (IBM, Armonk, NY, USA). Data on NTS A2 neuron activation during Pavlovian appetitive conditioning were analyzed using mixed ANOVA where the between subject factor was genotype and within subject factors were treatment and CS. When ANOVA identified a significant interaction, separate analyses were performed for each genotype. All other behavioral data were analyzed using repeated measures ANOVA. When ANOVA identified a significant interaction, paired t-tests were performed to determine differences between groups.

## Results

### Foot shock stress reduced cue-induced appetitive behavior

**Fig. 1:**
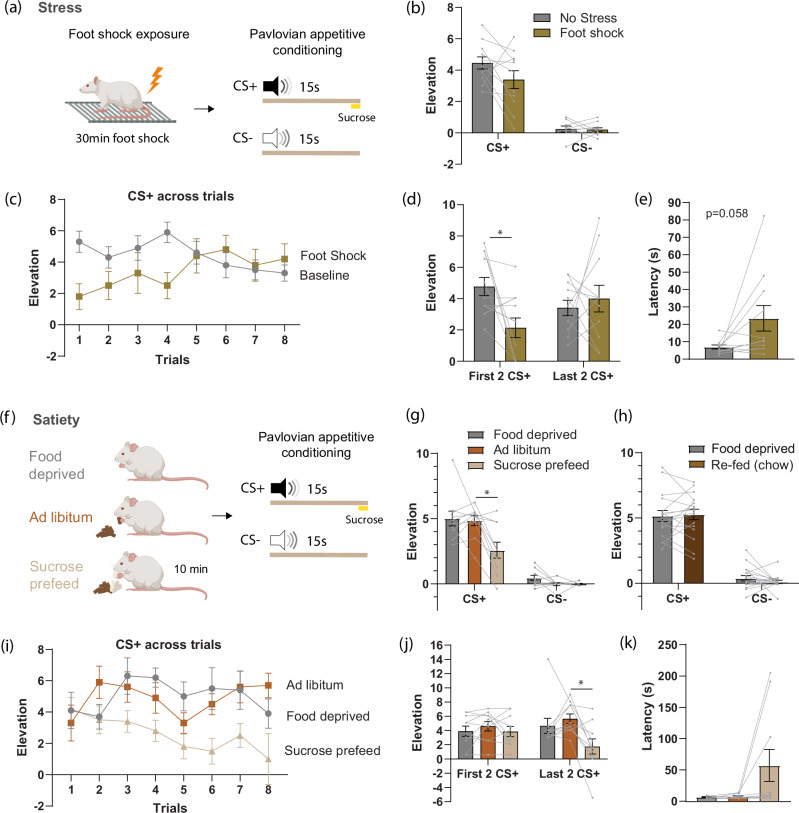
Stimuli that suppress cue-induced appetitive behavior. **a** Schematic of experimental design examining the effects of foot shock stress on cue-induced appetitive behavior. Rats received foot shocks for 30 min immediately prior to being tested in the Pavlovian appetitive conditioning paradigm. **b** There was no effect of foot shock stress on elevation scores during CS+ or CS-. or **c** CS+ elevation across trials, however, there was a significant stress x trial interaction. **d** Elevation scores during the first 2 CS + , but not last 2 CS+ was significantly lower following foot shock stress compared to the no stress control. **e** There was no effect of foot shock stress on latency to respond to CS+, despite a trend towards statistical significance. **f** Schematic of experimental design for examining the effects of sucrose prefeed/satiety on cue-induced appetitive behavior. **g** Compared to animals under food deprivation and ad libitum conditions, animals pre-fed with sucrose significantly suppressed elevation scores during CS+ but not CS-. **h** Re-feeding animals with chow did not impact CS elevation scores. **i** CS+ elevation across trials. There was a main effect of trial and condition x trial interaction. **j** Sucrose prefeed significantly reduced elevation during last 2 CS+ but not first 2 CS + . **k** There was no effect of sucrose prefeed on latency to respond to CS + .

The effect of stress on cue-induced appetitive behavior was assessed by delivering mild foot shocks prior to Pavlovian appetitive conditioning task. Results showed a significant main effect of CS (F_(1,10)_ = 158.633, *P* < 0.001), where CS+ elevation scores were higher than CS-. There was no effect of stress, although there was a trend towards significance (F_(1,10)_ = 3.959, *P* = 0.075). There was also no stress x CS interaction (F_(1,10)_ = 2.152, *P* = 0.17) (Fig. 1b).

To determine whether the effect of stress was transient, we examined CS+ elevation across trials. While there was no effect of stress (F_(1,10)_ = 3.227, *P* = 0.10) or trial (F_(7,70)_ = 0.561, *P* = 0.79), there was a significant stress x trial interaction ((F_(7,70)_ = 2.867, *P* < 0.05) (Fig. 1c), indicating possible differences in CS+ responses across trials between stress and no stress conditions. Further analysis examining the first two trials and last two trials revealed that foot shock stress significantly reduced CS+ elevation during the first 2 trials (*t* = 2.807, *P* < 0.05) but not the last 2 trials (*t* = −0.534, *P* = 0.605) (Fig. 1d), thus demonstrating a transient effect of foot shock on cue-induced appetitive behavior. There was also a trend towards an increase in latency to respond to CS+ following foot shock stress (F_(1,10)_ = 4.595, *P* = 0.058) (Fig. 1e).

Together, these results suggest that foot shock stress transiently reduced cue-induced appetitive behavior.

### Prefeed with sucrose reduced cue-induced appetitive behavior

Rats were subjected to 3 conditions (food deprived, ad libitum, sucrose prefeed) and the impact of these conditions on cue-induced appetitive behavior was assessed. Repeated measures ANOVA revealed a main effect of condition (F_(2,18)_ = 8.544, *P* < 0.01), CS (F_(1,9)_ = 126.362, *P* < 0.01) and condition x CS interaction (F_(2,18)_ = 7.069, *P* < 0.05). Pairwise comparisons showed that sucrose prefeed significantly reduced appetitive behavior to CS + , but not CS-, when compared to responses during ad libitum condition (*P* < 0.01) (Fig. 1g). There was no difference in CS+ induced appetitive behavior between ad libitum fed and food deprived conditions, suggesting that outcome specific satiety, rather than the energy status per se, affects cue-induced appetitive behaviors. To ensure that the effects observed were due to outcome specific satiety and not overall satiation, another group of rats that were previously trained on Pavlovian appetitive conditioning was food-deprived overnight and satiated with standard chow prior to being tested on Pavlovian appetitive conditioning task. Here, we did not observe any differences in CS elevation scores between groups (t_(16)_ = −0.314, *P* = 0.76) (Fig. 1h), indicating an outcome specific satiety effect on cue-induced appetitive behavior.

Further analysis of CS+ elevation scores across trials under deprivation, ad libitum and prefeed conditions were performed to determine whether the effect of condition on appetitive behavior differed across trials. Repeated measures ANOVA showed a main effect of condition (F_(2,18)_ = 8.162, *P* = 0.01) and condition x trial interaction (F_(14,126)_ = 1.845, *P* < 0.05) without any effect of trial (F_(7,63)_ = 1.112, *P* = 0.37) (Fig. 1i), suggesting that differences in condition affect appetitive behaviors across trials. We then split the data into the first 2 trials and the last 2 trials. In contrast to the effects of stress observed from the previous experiment, there was no group difference in appetitive behaviors during the first 2 CS+ trials (F_(2,18)_ = 0.667, *P* = 0.53). However, there was a main effect of condition on the last 2 CS+ trials (F_(2,18)_ = 6.141, *P* < 0.01) where rats in the prefeed condition showed significantly lower elevation scores compared to the ad libitum condition (*P* < 0.05) (Fig. 1j). There was no effect of condition on latency to enter magazine during CS+ (F_(2,18)_ = 3.966, *P* = 0.08) (Fig. 1k). Overall, these results show that outcome specific satiety, induced by sucrose prefeeding, reduced cue-induced appetitive behavior.

### Inhibition of NTS neurons attenuated foot shock stress-induced suppression of cue-induced appetitive behavior

**Fig. 2:**
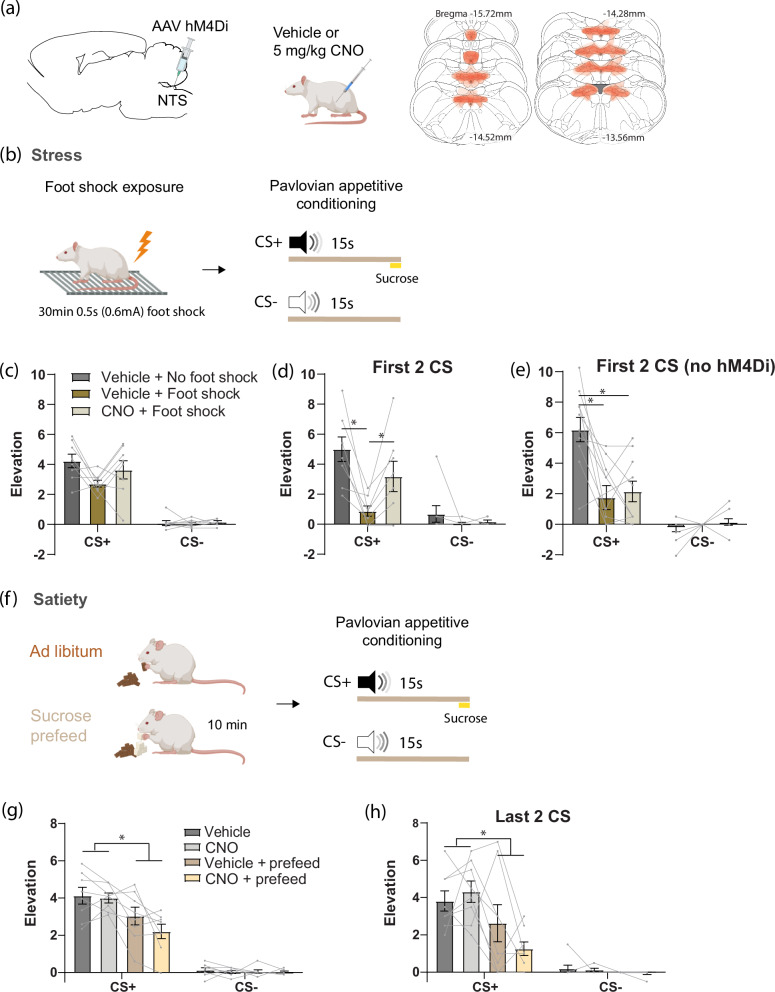
Effects of NTS inhibition on stress- and satiety-induced suppression of cue-induced appetitive behavior. **a** Schematic of NTS inhibition and hM4Di mCherry expression throughout NTS. **b** Schematic of experimental design for foot shock stress-induced suppression of cue-induced appetitive behavior. **c** Foot shock stress did not suppress cue-induced appetitive behavior and CNO had no effect. **d** Analysis of the first 2 CS showed a condition x CS interaction such that foot shock significantly reduced CS+ elevation, but not CS- elevation, and CNO attenuated this suppression. **e** In animals that did not express hM4Di in the NTS, there was a condition x CS interaction where foot shock and foot shock with CNO treatment significantly suppressed CS + , but not CS- elevation. **f** Schematic of experimental design for sucrose prefeed/satiety-induced suppression of cue-induced appetitive behavior. **g** Prefeed significantly suppressed CS + , but not CS- elevation (prefeed x CS interaction), without any effect of CNO. **h** Analysis of the last 2 CS also showed a main effect of prefeed on CS+ elevation, without any effect of CNO.

Given that NTS is a key brain region that integrates interoceptive and viscerosensory information to modulate behavior, we then examined whether NTS neurons mediates stress-induced suppression of cue-induced appetitive behavior. To do so, we inhibited NTS neural activity using the inhibitory DREADD hM4Di and determined whether it attenuated the effects of foot shock stress on cue-induced appetitive behavior. There were 3 conditions: Vehicle + no foot shock, Vehicle + foot shock and CNO + foot shock. We showed a significant main effect of CS (F_(1,7)_ = 109.069, *P* < 0.001) but a trend towards significance for condition (F_(2,14)_ = 2.998, *P* = 0.08) and condition x CS interaction (F_(2,14)_ = 3.195, *P* = 0.07) (Fig. 2c). Given that the effects of foot shock stress on cue-induced appetitive behavior were more pronounced during the first 2 CS + , we examined the effects of NTS neuron inhibition during the first 2 CS. Here, results showed a main effect of condition (F_(2,14)_ = 7.685, *P* < 0.05), CS (F_(1,7)_ = 24.104, *P* < 0.01) and condition x CS interaction (F_(2,14)_ = 5.32, *P* < 0.05). Subsequent analysis on the first 2 CSs showed that there was a main effect of condition on CS+ (F_(2,14)_ = 7.587, *P* < 0.05) but not CS- (F_(2,14)_ = 1.122, *P* = 0.35), where rats receiving Vehicle + foot shock showed significant lower CS+ elevation compared to Vehicle + no foot shock (*P* < 0.01) and CNO + foot shock (*P* < 0.05) (Fig. 2d). There was no difference in CS+ elevation between Vehicle + no foot shock and CNO + foot shock conditions, thus indicating that inhibition of NTS attenuated foot shock-induced suppression of cue-induced appetitive behavior.

To ensure that the effects were specific to NTS inhibition, a separate group of animals without hM4Di expression in the NTS was tested under the same conditions. Analysis of the first 2 CSs showed that whilst there was a main effect of condition (F(2,18) = 10.767, *P* < 0.001), CS (F(1,9) = 38.597, *P* < 0.001) and condition x CS interaction (F(2,18) = 12.282, *P* < 0.001), rats subjected to foot shock stress, regardless of whether they received vehicle or CNO treatment, showed reduced CS+ elevation relative to no foot shock controls (*P* < 0.05) (Fig. 2e), further confirming the specificity of NTS inhibition on foot shock stress-induced suppression of cue-induced appetitive behavior. Together, results indicate that NTS neurons are required for the suppression of cue-induced appetitive behavior induced by foot shock stress.

### Inhibition of NTS neurons had no effect on prefeed-induced suppression of cue-induced appetitive behavior

We also examined whether NTS neurons mediate the effects of a sucrose prefeed on cue-induced appetitive behavior. To do so, we inhibited NTS neural activity and subjected rats to either the sucrose prefeed or ad libitum condition and measured their appetitive behavior in the Pavlovian appetitive conditioning task. Consistent with our previous finding, there was a main effect of prefeed (F_(1,7)_ = 29.484, *P* < 0.001), CS (F_(1,7)_ = 125.741, *P* < 0.001), but no effect of treatment (F_(1,7)_ = 3.375, *P* = 0.109). There was an interaction between CS and prefeed (F_(1,7)_ = 20.447, *P* < 0.01), indicating that prefeed suppressed CS+ elevation (F_(1,7)_ = 26.517, *P* < 0.01) but not CS- (F_(1,7)_ = 0.215, *P* = 0.657) (Fig. 2g). Thus, these results suggest that NTS does not mediate the suppression of cue-induced appetitive behavior by sucrose prefeed.

Since we showed that the effect of prefeed was more pronounced towards the end of the session (Fig. 1j), we examined whether there was an effect of NTS inhibition on prefeed-induced suppression in appetitive behavior during the last 2 CSs. As expected, there was a main effect of prefeed (F(1,7) = 9.993, *P* < 0.05), CS (F(1,7) = 71.258, *P* < 0.001) and CS x prefeed interaction (F(1,7) = 7.074, *P* < 0.05). There was however no effect of treatment (F(1,7) = 0.727, P = 0.422), treatment x prefeed interaction (F(1,7) = 1.629, *P* = 0.243) or treatment x prefeed x CS interaction (F(1,7) = 2.166, *P* = 0.185) (Fig. 2h), indicating that inhibition of NTS had no effect on the suppression of appetitive behavior from prefeed.

These results show that, unlike stress, NTS neurons are not required for sucrose prefeed-induced suppression of cue-induced appetitive behavior.

### Activation of NTS A2 neurons reduced cue-induced appetitive behavior

Given that NTS A2 neurons are activated by foot shock stress [7], we then examined whether NTS A2 neurons are sufficient to suppress cue-induced appetitive behavior. We used chemogenetics in a TH Cre rat, previously validated by our lab [11], to activate NTS A2 neurons.

**Fig. 3:**
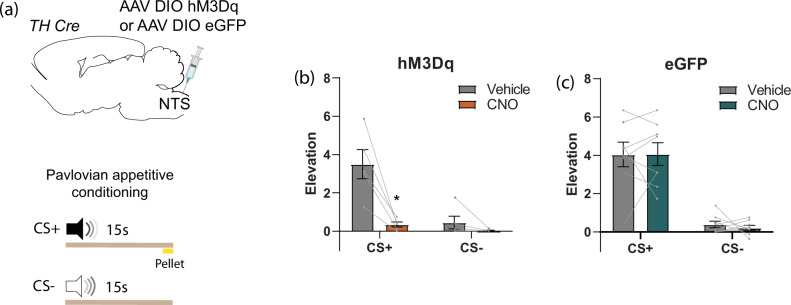
Effects of NTS A2 stimulation on cue-induced appetitive behavior. **a** Schematic of experimental design. **b** In TH Cre animals injected with hM3Dq to the NTS, there was a treatment x CS interaction where CNO administration significantly suppressed elevation during CS+ but not CS-. **c** In animals injected with eGFP to the NTS, CNO administration had no effect on CS elevation.

Results showed a main effect of treatment (F(1,11) = 10.709, P < 0.05), CS (F(1,11) = 63.349, *P* < 0.01), treatment x genotype (F(1,11) = 9.204, *P* < 0.05), CS x genotype (F(1,11) = 9.114, *P* < 0.05), treatment x CS (F(1,11) = 7.771, *P* < 0.05), treatment x CS x genotype (F(1,11) = 10.238, *P* < 0.01). In hM3Dq rats, but not eGFP rats, there was a main effect of treatment (F(1,4) = 12.992, *P* < 0.05), CS (F(1,4) = 30.890, *P* < 0.01), treatment x CS interaction (F(1,4) = 33.374, P < 0.01) (Fig. 3b, c). Further analysis in hM3Dq rats revealed that CNO significantly reduced CS+ elevation when compared to vehicle (F(1,4) = 19.516, *P* < 0.05) but not CS- (F(1,4) = 1.908, *P* > 0.05) (Fig. 3b). Thus, like foot shock stress, activation of NTS A2 neurons suppressed cue-induced appetitive behavior.

### Activation of NTS A2 neurons reduced VTA dopamine neural activity during CS + 

**Fig. 4:**
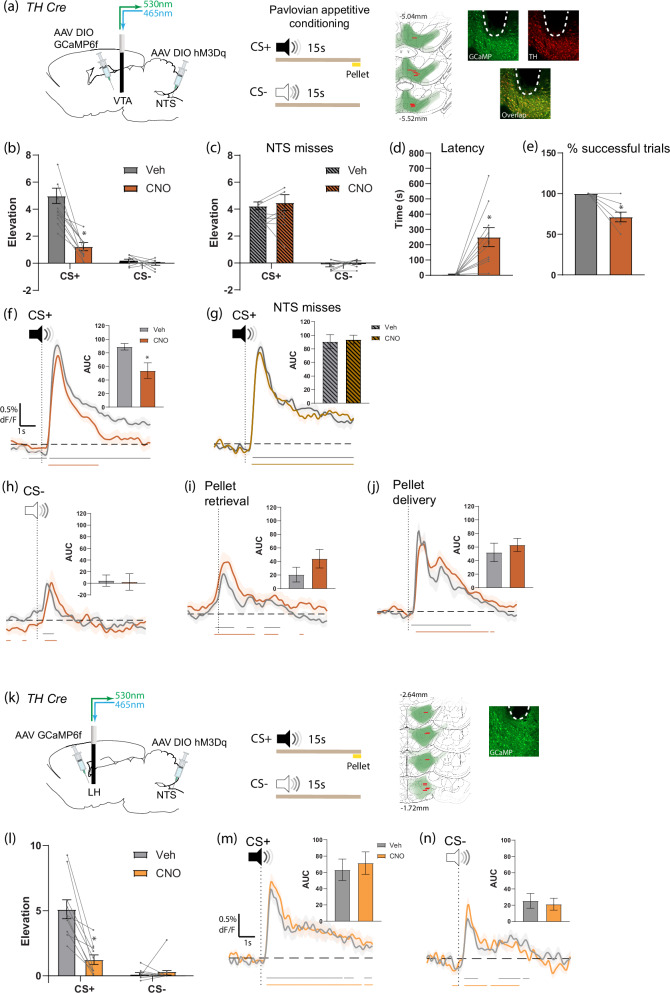
Effects of NTS A2 neuron stimulation on cue-induced appetitive behavior and VTA dopamine (a–j) and LH (k–n) neuron activity. **a** Schematic of experimental design, viral and cannula placements in the VTA, and representative images of VTA cannula, GCaMP and TH expression. **b** CNO administration significantly suppressed CS+ elevation. **c** In animals without hM3Dq in the NTS, CNO administration had no effect on CS+ elevation. **d** CNO increased latency to respond to CS+ and **e** reduced the % of successful trials. **f** CS+ increased VTA dopamine neuron Ca^2+^ transients and CNO significantly attenuated the increase. Area under the curve (AUC) analysis showed that CNO reduced VTA dopamine neuron activity. **g** In animals without hM3Dq in the NTS, there was no difference in VTA dopamine neuron Ca^2+^ transients between Vehicle and CNO treatment. CNO also had no effect on VTA dopamine neuron Ca^2+^ transients following (**h**) CS- presentation, **i** pellet delivery or (**j**) pellet retrieval. **k** Schematic of experimental design, viral and cannula placements in the LH, and representative image of LH cannula and GCaMP expression. **l** CNO administration significantly suppressed CS+ elevation. LH neuron Ca^2+^ transients increased following **m** CS+ and **n** CS- presentations but CNO had no effect on LH neuron Ca^2+^ transients. Colored lines below transients indicate periods of statistical significance for each transient when 95% confidence interval does not contain 0 for at least 0.5 s.

To identify a mechanism through which NTS A2 neurons suppress cue-induced appetitive behavior, we examined the effects of NTS A2 neuron stimulation on VTA dopamine neural activity during Pavlovian Appetitive Conditioning. As previously reported, stimulation of NTS A2 neurons significantly suppressed cue-induced appetitive behaviors, with a reduction in CS+ (*t* = 5.743, *P* < 0.001) but not CS- elevation scores (t = 0.937, *P* = 0.373) (Fig. 4b). These effects were specific to hM3Dq DREADD because animals without hM3Dq expression in the NTS failed to show any effects of treatment (F_(1,7)_ = 0.569, *P* = 0.475) or treatment x CS interaction (F_(1,7)_ = 0.023, *P* = 0.883) (Fig. 4c). We then analyzed VTA dopamine neuron activity transients by calculating AUC from event onset for 7 seconds. Results showed a significant decrease in CS + AUC following NTS A2 neuron activation (t = 3.270, *P* = 0.01) (Fig. 4f), but not CS- (*t* = 0.168, *P* = 0.846) (Fig. 4h), pellet delivery (*t* = −952, *P* = 0.366) (Fig. 4j) or pellet retrieval (*t* = −1.421, *P* = 0.189) (Fig. 4i). Consistent with the lack of effects of CNO on cue-induced appetitive behavior in rats without NTS hM3Dq expression, there was also no difference in CS + AUC (*t* = −0.337, *P* = 0.746) whether the rat was injected with Vehicle or CNO (Fig. 4g). We also examined whether foot shock stress emulates the effects of NTS A2 neuron stimulation on cue-evoked VTA dopamine neuron activity. Results showed that foot shock stress also suppressed VTA dopamine neuron activity during CS+ (*t* = 2.739, *P* < 0.05), but not during CS- (*t* = −0.330, *P* = 0.748) (Supplementary Fig. 1b). Together, these results indicate that stimulation of NTS A2 neurons, similar to foot shock stress, suppressed CS+ evoked VTA dopamine neuron activity.

### Activation of NTS A2 neurons had no effect on LH neural activity during CS + 

To determine whether the effects of NTS A2 stimulation are specific to VTA dopamine neural activity, we also examined LH neural activity during Pavlovian Appetitive Conditioning. Here we showed that while activation of NTS A2 neurons suppressed cue-induced appetitive behavior (*t* = 5.181, *P* < 0.001) (Fig. 4l), LH neural activity during CS+ (*t* = −1.228, *P* = 0.251) (Fig. 4m) or CS- (*t* = 0.664, *P* = 0.523) (Fig. 4n) was not different between treatments. Thus, NTS A2 neuron activation did not affect CS-evoked changes in LH neural activity, suggesting that the effects of NTS A2 neurons are not mediated via the LH.

## Discussion

This study examined the role of NTS in modulating cue-induced feeding behaviors under conditions of stress and satiety. While both acute stress and outcome specific satiety suppressed cue-induced appetitive behavior, NTS neurons selectively mediated the suppression caused by acute stress. Further analysis of NTS neural phenotypes revealed that, like foot shock stress, activation of NTS A2 neurons suppressed cue-induced appetitive behavior, and this was accompanied by an attenuation of cue-evoked VTA dopamine neural activity, without any effect on LH neural activity. Collectively, outcomes from this study suggest that NTS neurons contribute to stress-induced suppression of cue-induced appetitive behaviors and NTS A2 neurons do so, putatively by attenuating cue-evoked VTA dopamine neural activity.

We showed that NTS is necessary for the inhibitory effect of stress, but not outcome specific satiety, on cue-induced appetitive behavior. This selective involvement aligns with prior evidence that NTS neurons are activated by psychogenic stressor (e.g., foot shock) [7, 18–20] and contribute to stress-induced hypophagia [9, 21], but are not required for the processing of sensory-specific satiety [22]. Instead, other brain regions such as the orbitofrontal cortex and nucleus accumbens, which are implicated in sensory-specific satiety [22, 23], may contribute to the inhibitory effects of satiety on cue-induced appetitive behavior. Interestingly, a recent study showed that brainstem GLP-1 receptor signaling is required for sensory specific satiety [24]. Whether the effects are specific to the NTS are unclear but this finding underscores the need to further understand the mechanisms of sensory specific satiety. Nonetheless, our findings highlight a dissociation in the neural circuits underlying stress and satiety, with NTS neurons selectively modulating stress-related suppression of cue-driven appetitive behavior.

To identify the NTS neural phenotype involved in cue-induced feeding, we selectively targeted NTS A2 neurons, a major stress-responsive population known to suppress food intake [7, 11]. We showed for the first time that activation of NTS A2 neurons significantly suppressed cue-induced appetitive behavior, mimicking the acute effects of foot shock stress in the absence of a stressor. This suppression in cue-induced appetitive behavior is unlikely due to overall behavioral suppression as we previously showed that activation of NTS A2 neurons has no effect on nausea/malaise, locomotor activity or anxiety-like behavior in an elevated plus maze test [11]. Nonetheless, we showed that chemogenetic activation of NTS A2 neurons increases plasma corticosterone levels [11], which supports the idea that NTS A2 neurons contribute to stress-responsive pathways that regulate cue-induced appetitive behaviors. Consistent with this, central infusion of corticotropin-releasing factor (CRF), a key mediator of the stress response, activates NTS A2 neurons [25], further implicating these neurons in stress-related modulation of feeding behavior. In addition to stress, neuropeptides such as oxytocin and GLP-1, which also activate A2 neurons, act within the NTS to reduce cue-induced food seeking [26–28]. This suggests that NTS A2 neurons may integrate multiple inhibitory signals, including stress, to modulate cue-induced appetitive behavior.

To determine the underlying mechanism, we examined VTA dopamine neurons, which are critical for encoding food-predictive cues and driving cue-induced approach behavior [29, 30]. We found that stimulation of NTS A2 neurons attenuated food cue-evoked VTA dopamine neuron activity. Importantly, the attenuated dopamine neuron response was specific to the food cue as stimulation of NTS A2 neurons had no effect on VTA dopamine neuron activity during CS-, pellet delivery or retrieval. Furthermore, foot shock also suppressed cue-induced VTA dopamine neuronal activity in a way similar to the effect seen with stimulation of NTS A2 neurons. This provides further support for the link between NTS A2 neurons and stress.

Given the positive relationship between cue-evoked VTA dopamine neuron activity and cue-induced approach/licking behavior [29], our findings suggest that stress, likely through NTS A2 neurons, suppresses cue-induced appetitive behavior by dampening cue-evoked VTA dopamine neuron activity. Interestingly, this pattern of suppression mirrors the effects of several appetite inhibitory peptides, including leptin, oxytocin and GLP-1, which also reduce cue-evoked VTA dopamine neuron activity and suppress cue-induced feeding behaviors [29–32]. These parallels suggest that stress, NTS A2 neurons and appetite regulatory peptides converge on a shared downstream mechanism involving VTA dopamine neurons to suppress cue-driven appetitive behaviors.

Given that LH receives input from NTS A2 neurons [11] and inhibition of VTA-projecting LH neurons suppresses cue-induced appetitive behavior [33], we also examined whether activation of NTS A2 neurons affects LH neural activity during Pavlovian appetitive conditioning. In contrast to the effects on VTA dopamine neurons, activation of NTS A2 neurons had no effect on cue-evoked LH neural activity. This finding was unexpected given that electrophysiological studies show that stimulation of the ventral noradrenergic bundle (including NTS A2 neurons) inhibits LH neurons [34] and bath application of noradrenaline reduces excitability of LH orexin neurons [35, 36]. The lack of effect of NTS A2 neurons on LH neural activity could be due to several reasons. First, NTS A2 neurons also co-express glutamate, which could activate LH neurons. This could cancel out the inhibitory effects of noradrenaline on LH neurons. However, whether and how glutamate and noradrenaline act in concert in the LH is unclear. Second, LH is highly heterogenous [37], which comprises of not only orexin neurons but also melanin concentrating hormone (MCH), GABAergic galanin and neurotensin neurons. While LH orexin, MCH and GABA neurons are implicated in cue-induced feeding [38–40], LH neurotensin neurons do not respond to food-predictive cues [41], which suggests functional differences amongst LH neuron populations. Thus, more selective targeting of LH neuron population may be required to determine whether different populations of LH neurons are affected by NTS A2 neuron stimulation. Nonetheless, our findings indicate that the effects of NTS A2 neurons on cue-evoked neural activity is specific to VTA dopamine neurons.

The circuits mediating the effects of NTS A2 neurons on cue-induced appetitive behavior and VTA dopamine neuron activity remain to be determined. It is possible that NTS A2 neurons that project to the VTA to suppress VTA dopamine neuron activity and cue-induced appetitive behavior. However, this projection is sparse [11, 42], and its functional significance is still unclear. It is also possible that the effects are mediated via a polysynaptic pathway. One potential mediating brain region is the parabrachial nucleus (PBN) as it is a main relay area for sensory information from NTS to the forebrain. Previous studies show that PBN calcitonin gene-related peptide (CGRP) neurons receive inputs from the NTS to regulate feeding [43] and also project to the VTA to reduce food-seeking [44]. Another possible mechanism is via the NAc, which primarily receives noradrenaline input from NTS A2 neurons [16] and also projects to the VTA to control context-induced relapse [45]. In a recent study, we showed that noradrenaline binding in the NAc declines during food cue presentation and conditioned approach [17], suggesting that the NTS A2 → NAc pathway could contribute to cue-induced feeding behaviors. Future studies are required to examine the contribution of these circuits in mediating the effects of NTS A2 neurons on cue-induced appetitive behaviors.

While this study focused on NTS A2 neurons, we acknowledge that other NTS populations could also contribute to the regulation of cue-induced feeding during stress. For example, NTS preproglucagon (PPG) neurons are also activated by stressors and are implicated in stress-induced hypophagia [9, 25, 46]. They project to the VTA and activation of VTA GLP-1 receptors modulates the excitability of VTA neurons [32, 47]. Systemic GLP-1 analogue administration also suppresses cue-evoked licking behavior and dampens VTA dopamine neural activity during food cue presentation [29], paralleling the effects observed with NTS A2 neuron stimulation. Although it is unknown whether NTS PPG neurons are activated by foot shock, other psychogenic stressors, such as restraint, recruit both GLP-1 and A2 neurons [7, 46]. Thus, we cannot exclude the possibility that NTS PPG neurons may also contribute to the effects of stress on cue-induced feeding behavior.

There are several limitations to the present study. First, we used foot shock as a stressor, which raises the possibility that rats also experienced pain. However, this is unlikely given that there were no typical nociceptive behaviors such as paw-licking during the foot shock sessions. To minimize this potential confound, future studies could consider using other psychogenic stressors such as restraint, to confirm the role of NTS/NTS A2 neurons in mediating the effects of stress on cue-induced appetitive behavior. Second, this study used males only, thus whether the same effects are observed in females is unclear. Current studies show sex differences in response to stress, but findings are mixed: novelty stress suppresses food intake more in females [48], while restraint stress suppresses food intake in males but not females [49]. Moreover, females exposed to adolescent or adult foot shock stress show higher cue-induced motivated behavior, compared to males [3]. These discrepancies may reflect differences in stressor type and feeding paradigm used. There are also sex differences in NTS PPG neuron activation where activation of NTS PPG neurons suppresses food intake and increases anxiety-like behavior in females but not in males [25]. Therefore, future studies should determine whether females exhibit similar effects of stress, satiety and NTS manipulation on cue-induced appetitive behaviors as those observed in male rats in the present study.

In conclusion, we showed that NTS neurons are necessary for foot shock stress, but not outcome specific satiety, to suppress cue-induced appetitive behavior. This effect is in part mediated via NTS A2 neurons, as activation of these neurons suppressed conditioned approach and attenuated cue-evoked VTA dopamine neuron, but not LH neuron activity. Together, findings from this study highlight that, while both stress and satiety reduce cue-induced feeding, they engage distinct neural circuits, with the effects of stress being mediated via an NTS-dependent mechanism.

## Supplementary information

Supplementary Figure 1

Supplementary materials and methods

## Data Availability

Data from this study will be made available upon request.
