# Supplementary material for: Nucleus of the solitary tract regulation of cue-induced appetitive behaviors via midbrain dopamine neurons
Source: Neuropsychopharmacology. 2026 Apr 22;51(10):1826–35. doi: 10.1038/s41386-026-02417-y (PMC13486705; doi:10.1038/s41386-026-02417-y)
Supplement: Supplementary file 1 [file 41386_2026_2417_MOESM1_ESM.docx]

Supplementary Figure 1: Effects of foot shock on VTA dopamine neuron activity. (a) Schematic of experimental design. Foot shock significantly attenuated VTA dopamine neuron Ca^2+^ transients following (b) CS+ but not (c) CS-, as indicated by the AUC analysis. Colored lines below transients indicate periods of statistical significance for each transient when 95% confidence interval does not contain 0 for at least 0.5 s.
