# Supplementary material for: Nucleus of the solitary tract regulation of cue-induced appetitive behaviors via midbrain dopamine neurons
Source: Neuropsychopharmacology. 2026 Apr 22;51(10):1826–35. doi: 10.1038/s41386-026-02417-y (PMC13486705; doi:10.1038/s41386-026-02417-y)
Supplement: Supplementary file 2 [file 41386_2026_2417_MOESM2_ESM.docx]

**Supplementary materials and methods**

*Surgery*

For experiments using DREADDs, rats were infused with either AAV-hSyn-hM4Di or AAV-hSyn-DIO-hM3Dq (200nl each side) bilaterally to the NTS (Occipital AP +1.7 mm, ML ±0.5 mm, Skull DV –8.6 mm, or Occipital AP +1.9 mm, ML ±0.5 mm, Skull DV –9.3 mm, 15˚ anterior-posterior). For pathway specific DREADDs experiments, rats were injected with AAV-hSyn-DIO-hM3Dq or AAV-hSyn-DIO-mCherry (200nl each side) bilaterally to the NTS and a bilateral infusion cannula (1.0mm c-c; Plastics One Inc) implanted targeting the VTA (Bregma AP −5.9 mm, ML ±0.7 mm, DV −6.7 mm, 2mm projection tip used for infusions). For fibre photometry experiments, rats were unilaterally injected with AAV-CAG-FLEX-GCaMP6f (400 nL) targeting the VTA (Bregma AP −5.9 mm [males]/-5.7 [females], ML ±0.7 mm, DV −8.7 mm) or the LH (Bregma AP -2.5 mm, ML ±2.0 mm, DV -8.8 mm) and then implanted with a fiber optic cannula in the target area. Brain coordinates were based on [1].

*Viral vectors*

pAAV-hSyn-hM4D(Gi)-mCherry was a gift from Bryan Roth (Addgene viral prep # 50475-AAV5; http://n2t.net/addgene:50475 ; RRID:Addgene_50475),

pAAV5-hSyn-DIO-hM3D(Gq)-mCherry was a gift from Bryan Roth (Addgene viral prep # 50474-AAV5; http://n2t.net/addgene:50474 ; RRID:Addgene_50474), pAAV-EF1a-double floxed-hChR2(H134R)-EYFP-WPRE-HGHpA was a gift from Karl Deisseroth (Addgene viral prep # 20298-AAV5; http://n2t.net/addgene:20298 ; RRID:Addgene_20298), pAAV-Ef1a-DIO EYFP was a gift from Karl Deisseroth (Addgene viral prep # 27056-AAV5; http://n2t.net/addgene:27056 ; RRID:Addgene_27056), pAAV.CAG.Flex.GCaMP6f.WPRE.SV40 was a gift from Douglas Kim & GENIE Project (Addgene viral prep # 100835-AAV5 ; http://n2t.net/addgene:100835 ; RRID:Addgene_100835).

*Apparatus*

All procedures were conducted in a standard MedAssociates rat conditioning chamber with grid floors. The presentation of stimuli was controlled by MED-PC (MedAssociates).

*Pavlovian appetitive conditioning*

Rats were presented with two auditory cues (tone and clicker) and were trained to associate one auditory cue with the delivery of a sucrose pellet (CS+) and another cue without (CS-). The CSs were counterbalanced such that one group of rats received tone as CS+ and clicker as CS- while the other group received clicker as CS+ and tone as CS-. Each session consisted of a randomized order of eight 15s CS+ and eight 15s CS-, with an intertrial interval (ITI) of four minutes. Sucrose pellets were used as the food reward and its delivery co-terminated with the presentation of CS+. Training was at least 5 days and testing begun after rats have learnt the task.

*Fiber photometry*

The patch cord was connected to LEDs with excitation wavelengths 465 nm (Ca^2+^ dependent) and 405 nm (isosbestic). Light from LEDs was controlled by a LED driver and channeled into the patch cord and cannula via a minicube. Fluorescence signals detected were amplified and measured by a photoreceiver, demodulated and low pass filtered (3Hz) through Synapse software and RZ5P system. This system was also connected to MED-PC program to allow for timestamp of behavioral events (e.g., CS presentations, magazine entries) and signals in real time. Light intensity from the patch cord was >5µW across sessions. Fiber photometry data were analyzed using custom MATLAB scripts. Isosbestic 405 nm signal was regressed onto Ca^2+^ dependent 465 nm signal to generate a fitted 405 nm signal. dF/F was generated by subtracting the fitted 405 nm signal from 465 nm signal and then divided by fitted 405 nm. Signals were low pass (3Hz) and high pass (90s) filtered. Bootstrap confidence interval analysis was performed where dF/F waveforms were resampled with 1000 replacements to obtain a distribution of dF/F means. 95% confidence interval was set within the 2.5th and 97.5th percentiles of dF/F mean distribution and expanded by a factor of sqrt n/(n-1) to adjust for narrowness bias. Statistical significance is achieved when 95% confidence interval did not contain 0 for 0.5s [2]. Baseline transients were analyzed from -5s to 0 (event onset) and event transients were analyzed from 0 – 7s. Area under the curve for each event transient was calculated based on the trapezoidal method and differences between groups were analyzed using paired t-tests.

*Fluorescent immunohistochemistry*

Fluorescent immunohistochemistry was performed on a set of free-floating brain sections to verify viral expression. Sections were washed in PBS and blocked in 5% normal donkey serum. Primary antibodies, rabbit anti-GFP (1:1000, Thermo Fisher Scientific, catalog #11122, RRID: AB_221569) was used to label GCaMP6f in the VTA and rabbit anti-mCherry (1:1000, Abcam, RRID: ab167453) was used to label hM3Dq and hM4Di in the NTS. After overnight incubation with primary antibodies, sections were washed and incubated in secondary antibodies, donkey anti-rabbit 488 (1:500, Thermo Fisher Scientific, catalog #A-21206, RRID: AB_2535792) for VTA sections and donkey anti-rabbit 594 (1:500, Thermo Fisher Scientific, catalog #A-21207, RRID: AB_141637) for NTS sections. Sections were then mounted on slides and coverslipped with Fluoromount™ Aqueous Mounting Medium (Sigma Aldrich, United States). Slides were scanned using Axioscan slide scanner (Zeiss, Germany). GFP and mCherry expression were visualised using the software Zen 3.7 (Zeiss, Germany).

**References:**

1	Paxinos G, Watson C. The Rat Brain in Stereotaxic Coorinates*.* 6th ed. Academic Press: San Diego; 2007.

2	Jean-Richard-Dit-Bressel P, Clifford CWG, McNally GP. Analyzing Event-Related Transients: Confidence Intervals, Permutation Tests, and Consecutive Thresholds. Front Mol Neurosci. 2020;13:14.
